# Evolutionary and Regulatory Pattern Analysis of Soybean Ca^2+^ ATPases for Abiotic Stress Tolerance

**DOI:** 10.3389/fpls.2022.898256

**Published:** 2022-05-19

**Authors:** Jian Wang, Xujun Fu, Sheng Zhang, Guang Chen, Sujuan Li, Tengwei Shangguan, Yuanting Zheng, Fei Xu, Zhong-Hua Chen, Shengchun Xu

**Affiliations:** ^1^Central Laboratory, State Key Laboratory for Managing Biotic and Chemical Threats to the Quality and Safety of Agro-products, Zhejiang Academy of Agricultural Sciences, Hangzhou, China; ^2^Taizhou Seed Administration Station, Taizhou, China; ^3^College of Agriculture and Food Science, Zhejiang Agriculture and Forestry University, Hangzhou, China; ^4^School of Science, Western Sydney University, Penrith, NSW, Australia; ^5^Hawkesbury Institute for the Environment, Western Sydney University, Penrith, NSW, Australia

**Keywords:** abiotic stresses, Ca^2+^ ATPases, *Glycine max* L., phylogenetic analysis, stomatal regulation

## Abstract

P_2_-type Ca^2+^ ATPases are responsible for cellular Ca^2+^ transport, which plays an important role in plant development and tolerance to biotic and abiotic stresses. However, the role of P_2_-type Ca^2+^ ATPases in stress response and stomatal regulation is still elusive in soybean. In this study, a total of 12 P_2_-type Ca^2+^ ATPases genes (*GmACAs* and *GmECAs*) were identified from the genome of *Glycine max*. We analyzed the evolutionary relationship, conserved motif, functional domain, gene structure and location, and promoter elements of the family. Chlorophyll fluorescence imaging analysis showed that vegetable soybean leaves are damaged to different extents under salt, drought, cold, and shade stresses. Real-time quantitative PCR (RT-qPCR) analysis demonstrated that most of the *GmACAs* and *GmECAs* are up-regulated after drought, cold, and NaCl treatment, but are down-regulated after shading stress. Microscopic observation showed that different stresses caused significant stomatal closure. Spatial location and temporal expression analysis suggested that *GmACA8, GmACA9, GmACA10, GmACA12, GmACA13*, and *GmACA11* might promote stomatal closure under drought, cold, and salt stress. *GmECA1* might regulate stomatal closure in shading stress. *GmACA1* and *GmECA3* might have a negative function on cold stress. The results laid an important foundation for further study on the function of P_2_-type Ca^2+^ ATPase genes *GmACAs* and *GmECAs* for breeding abiotic stress-tolerant vegetable soybean.

## Introduction

Vegetable soybean (*Glycine max* L., also named “Maodou” in China and “edamame” in Japan) serves as a fresh vegetable and has higher nutritional content than grain- and oil-type soybean (Dong et al., 2014; Liu et al., 2017a). In recent years, the production of vegetable soybean has increased with the shift in consumer preference. Abiotic stresses such as low temperature, drought, and salinity have become important factors influencing the yield, taste, and nutritional quality of vegetable soybean (Xu et al., 2016). However, there is limited research work on the abiotic stress tolerance of vegetable soybean.

Cytosolic calcium concentration ([Ca^2+^]_cyt_) is a key cellular second messenger that plays a crucial role in plant growth, development, and stress response (Chen et al., 2010; Kudla et al., 2010; Seybold et al., 2014). In plants, guard cells integrate environmental and endogenous signals to regulate the aperture of stomatal pores and [Ca^2+^]_cyt_ oscillations are essential components of stomatal closure. [Ca^2+^]_cyt_ oscillation can regulate the closing of stomata through two mechanisms. Short-term calcium [Ca^2+^]_cyt_ oscillation can quickly close the stomata, while long-term [Ca^2+^]_cyt_ oscillation is controlled by a stable oscillation frequency, transient number, duration and amplitude that regulate stomatal closure (Allen et al., 2001; Fuglsang and Palmgren, 2021). In addition, calcium signaling can also participate in hormonal pathways to regulate stomatal movement under biotic and abiotic stresses (Tagliani, 2020; Thor et al., 2020; Hsu et al., 2021; Ou et al., 2022). Ca^2+^ has minimal physical mobility and is rarely transported between cells (Marschner, 2012). Therefore, it is necessary for plants to regulate intracellular Ca^2+^ concentration depending on the coordinated activities of pumps, channels, and co-transporters on the plasma membrane, tonoplast and membranes of different organelles (Bush, 1995; Sze et al., 2000; Tuteja and Mahajan, 2007). When external environmental stimuli induce the opening of Ca^2+^ channels, a rapid increase of [Ca^2+^]_cyt_ is perceived and decoded to give an appropriate response. Elevation of Ca^2+^ causes changes in the Ca^2+^ regulatory proteins and their targets, leading to the activation of downstream signaling events in different plant cell types (Clapham, 2007; Kudla et al., 2010; Bredow and Monaghan, 2018; Zhang et al., 2019).

Ca^2+^ transport in plant cells is mainly mediated by Ca^2+^ channels, Ca^2+^ antiporters, and Ca^2+^ pumps (White, 2000; Spalding and Harper, 2011; Wang et al., 2016; Demidchik et al., 2018). Ca^2+^ pumps belong to a large family of phosphorylated (P)-type ATPases located at the plasma membrane (PM), tonoplast, endoplasmic reticulum (ER), and Golgi (Table 1; Urbina et al., 2006; Hilleary et al., 2020). Generally, Ca^2+^ pumps are divided into ER-type Ca^2+^ ATPases (ECAs, P2A ATPases) and autoinhibited Ca^2+^ ATPases (ACAs, P2B ATPases) in plants, which have a higher affinity for Ca^2+^ transportation than Ca^2+^ channels and Ca^2+^ antiporters (Carafoli, 1991; Huda et al., 2013b; García Bossi et al., 2019). P2A ATPases serve housekeeping functions to load intracellular compartments with Ca^2+^ and/or Mn^2+^ whereas P2B ATPases are tightly regulated Ca^2+^-pumps that regulate cytoplasmic Ca^2+^ concentrations (subject to regulation by calmodulin and phosphorylation) that have the main role in signal transduction (Fuglsang and Palmgren, 2021). P_2B_-type pumps in plants contained an auto-inhibitory region that partly overlaps the calmodulin-binding domain at the N-terminal (Geisler et al., 2000b). Thus, calcium transport and ATP hydrolysis are inhibited in the absence of calmodulin, but this inhibition was eliminated by the binding of Ca^2+^ to the calmodulin-binding domain (Hwang et al., 2000; Lone et al., 2006; Lee et al., 2007).

**Table 1:** Summary of Ca^2+^-ATPase genes in *Glycine max* and the identity of Arabidopsis homologs.

Gene Name	Locus name	Gene Location	Protein length (aa)	MW (Da)	pI	GRAVY	Arabidopsis orthologs	Arabidopsis orthologs subcellular localization
GmACA1	GLYMA_04G045400	Chr4: 3622850..3633108: +	1,019	111255.82	5.92	0.181	ACA1	Endoplasmic reticulum^a^
GmACA2	GLYMA_01G193600	Chr1: 52814383..52820169: −	1,014	110604.22	5.72	0.173	ACA2	Endoplasmic reticulum^b^
GmACA8	GLYMA_15G1675001	Chr15: 14792771..14837236: +	1,082	117916.60	8.06	0.078	ACA8	Plasma membrane^c^
GmACA9	GLYMA_08G222200	Chr8: 038317..18058442: +	1,092	119274.47	6.39	0.030	ACA9	Plasma membrane^d^
GmACA10	GLYMA_17G057800	Chr17: 4370750..4397516: −	1,105	120782.81	7.81	0.091	ACA10	n.r.
GmACA11	GLYMA_19G136400	Chr19: 39763468..39774889: −	1,035	113482.71	5.75	0.166	ACA11	Vacuole membrane^e^
GmACA12	GLYMA_19G159900	Chr19: 42082444..42086717: −	1,069	118014.41	7.07	0.052	ACA12	Plasma membrane^f^
GmAC13	GLYMA_19G038600	Chr19: 5397711..5401490: −	1,029	113059.19	7.06	0.075	ACA13	n.r.
GmECA1	GLYMA_03G175200	Chr3: 38873627..38880295: +	1,060	116399.97	5.30	0.039	ECA1	Endoplasmic reticulum^g^
GmECA2	GLYMA_07G053100	Chr7: 4650513..4656886: −	1,073	118776.88	5.52	0.084	ECA2	n.r.
GmECA3	GLYMA_04G046700	Chr4: 3723235..3748189: +	1,001	109910.84	5.67	0.245	ECA3	Golgi^h^
GmECA4	GLYMA_19G175900	Chr19: 43587386..43593918: +	1,060	116530.04	5.22	0.031	ECA4	n.r.

PI, isoelectric point; MW, molecular weight; and GRAVY, Grand Average of Hydropathy; n.r.: Not reported.

a (Rahmati Ishka, 2015);

b (Hwang et al., 2000);

c (Bonza et al., 2000);

d (Schiøtt et al., 2004);

e (Lee et al., 2007);

f (Limonta et al., 2014);

g (Liang and Sze, 1998)

h (Li et al., 2008).

Ca^2+^ pumps are essential in many aspects of plant growth and development, including pollen tube growth, programmed cell death, and polarized tip growth in roots (Allen et al., 2001; Rajiv and Robinson, 2004; Kurusu et al., 2005; Young et al., 2006; Pandey et al., 2007; Monshausen et al., 2008; Hocking et al., 2017), as well as responses to abiotic stresses (e.g., shading, heat, cold, salt, drought, and osmotic stress), biotic stresses and symbiosis (e.g., fungi and bacteria; Lillo, 1994; Sanders et al., 1999; Rahman et al., 2016; Liu et al., 2017c; Yuan et al., 2018; Li et al., 2019). In Arabidopsis, pollen of loss-of-function mutant *aca9* displayed a reduced growth potential and a high frequency of aborted fertilization, resulting reduction in seed set (Schiøtt et al., 2004). Ca^2+^ ATPases are also available to be involved in the activation of salicylic acid (SA)-dependent programmed cell death regulated by AtACA4 and AtACA11 in Arabidopsis (Clapham, 2007; Yann et al., 2010). Ca^2+^-stimulated root growth and the detoxification of high Mn^2+^ are modulated by AtECA3 (Li et al., 2008). AtACA8 functions in the response to cold stress (Schiøtt and Palmgren, 2005), and AtACA8 and AtACA11 are involved in hypoxia stress (Wang et al., 2016). Overexpression of Arabidopsis *AtACA4* and *AtACA2* in yeast improved salt tolerance (Geisler et al., 2000a; Anil et al., 2007). In other plant species, overexpression of rice *OsACA6* in tobacco can effectively regulate ROS mechanism and proline synthesis and improve resistance to salt and drought stress (Huda et al., 2013a). The expression level of soybean *Ca^2+^-ATPase 1* (*SCA1*) was highly and rapidly induced by salt stress and a fungal elicitor (Chung et al., 2000). P_2B_-type Ca^2+^ ATPase loss-of-function mutants from the moss *Physcomitrella patens* exhibit susceptibility to salt stress (Enas et al., 2009).

Stomatal movement facilitates transpiration and photosynthesis, and actively regulates responses to biotic and abiotic stresses in plants (Melotto et al., 2008; Chen et al., 2017; Liu et al., 2017b; Wang and Chen, 2020). Stomatal closure stimuli such as abscisic acid (ABA), hydrogen peroxide, drought, salinity, cold, elevated external Ca^2+^ and elevated atmospheric CO_2_ (Gilroy et al., 1990; McAinsh et al., 1990, 2000; Allen et al., 2001; Sanders et al., 2002; Young et al., 2006; Chen et al., 2017). There is emerging evidence linking the Ca^2+^ ATPases with stomatal regulation. For instance, BONZAI1 (AtBON1) positively regulated the activities of AtACA8 and AtACA10. The *bon1*, *aca10*, and *aca8* knock-out mutants have defects in stomatal closure in response to environmental stimuli (Yang et al., 2017). However, the specific roles of other P_2_-type Ca^2+^ ATPases in stomatal function under external environmental stimulation have not yet been elucidated.

The objective of this study was to characterize P_2_-type Ca^2+^ ATPase genes in soybean by bioinformatics, physiological and molecular approaches. We hypothesized that *GmACAs* and *GmECAs* are key components of abiotic stress tolerance *via* the regulation of Ca^2+^ signaling in soybean. We analyzed potential family members of Ca^2+^ ATPases that may be involved in the regulation of abiotic stress and stomatal movement. We provide important information for further understanding of the biological functions of P_2_-type Ca^2+^ ATPases and utilization of P_2_-type Ca^2+^ ATPase genes for improving stress resistance and crop yield in soybean.

## Materials and Methods

### Plant Materials, Growth Conditions, and Abiotic Stress Treatments

For expression pattern analysis of P_2_-type Ca^2+^ ATPase genes under different abiotic stresses, the seeds of vegetable soybean cultivar “Zhexian 9” were placed in two rows (each row contained eight seeds) on double-layer filter papers (20 × 30 cm) with 3 cm away from the upper and lower edges. Then a layer of wet filter paper was covered on the seeds. The three-layer filter paper with seeds was rolled up from left to right and placed in a covered bucket (20 × 20 cm). Sterile water was added to the bucket every day. The bucket was placed in a light incubator under the conditions of 25°C/25°C (day/night), 12 h/12 h photoperiod (day/night) and 70% relative humidity for germination. Seedings with consistent germination were then transplanted into a pot filled with peat/vermiculite (2:1). Three seedlings in each pot were cultured in a light incubator in the same condition for germination and irrigated with water.

Two-week-old soybean seedlings (V4 stage, with three fully expanded leaves) were used to study the expression patterns of *GmACAs* and *GmECAs* after salt, drought, cold and shading stresses. Soybean seedlings under normal growth conditions were used as a control. In salt treatment, soybean seedlings were transferred to 250 mM NaCl solution for root soaking treatment (Yong et al., 2008). For drought treatment, seedlings were placed on dry filter papers in a light incubator (Chen et al., 2007; Chen et al., 2014). Potted seedlings were cultured in a light incubator at 4°C for cold treatment (Du et al., 2009). Moreover, potted seedlings were transferred to a light incubator with a photoperiod of 24 h (night) for shading treatment (Guimarães-Dias et al., 2012). The first fully unfolded true leaves of soybean were collected as samples at 0, 1, 6, 12, 24, and 48 h after treatment and immediately frozen in liquid nitrogen and stored at −80°C for RNA extraction. The control and each treatment had three biological repeats.

### Identification of the Soybean P_2_-Type Ca^2+^ ATPase Genes and Sequence Analysis

The genome sequence and gene annotation of *G. max*, *Ostreococcus tauri*, *Chlamydomonas reinhardtii*, *P. patens*, *Selaginella moellendorffii*, *Oryza sativa*, *Zea mays*, *Capsicum annuum*, and *Arabidopsis thaliana* were downloaded from the National Center for Biotechnology Information (NCBI) database.^1^ Hidden Markov Model (HMM) was used to predict soybean P_2_-type Ca^2+^ ATPase genes. The gene family sequence feature file E1-E2 ATPase (PF00122.20) was obtained from Pfam.^2^ PF00122.20 feature sequence file was alignment to soybean proteome database through Hmmer search online version 2.39.0^3^ with the E value at 10^−10^. The obtained protein sequences were searched for protein domains through the alignment Pfam-A database of the Hmmscan program. Sequences that did not contain family characteristic domains were deleted. BLASTp program^4^ was used for protein sequence alignment to search the genome sequence of *A. thaliana* and *O. sativa* for orthologs. Computation of the theoretical isoelectric point (pI) and molecular weight (Mw) of soybean P_2_-type Ca^2+^ ATPase proteins was performed using the compute pI/Mw online tool.^5^ Grand average of hydropathicity (GRAVY) was calculated using Protparam^6^ and ProtComp 9.0 was used to predict the sub-cellular localization.^7^

### Phylogenetic Analysis

Multiple amino acid sequences of identified P_2_-type Ca^2+^ ATPase genes were aligned using ClustalX2.1.0.0 software (Larkin et al., 2007). The phylogenetic trees comparing soybean and multiple species (*O. tauri*, *C. reinhardtii*, *P. patens*, *S. moellendorffii*, *O. sativa*, *Z. mays*, *C. annuum*, *A. thaliana*, and *G. max*) were utilized the neighbor-joining method and a graphical representation was produced with MEGA-X software and bootstrap analysis. The robustness of each node in the tree was determined using 1,000 bootstrap replicates with the pairwise deletion option. The phylogenetic trees comparing soybean and *A. thaliana* were also constructed with Mega-X by the parameters above. The MEME 5.0.5 online program (Bailey et al., 2015)^8^ was used for the identification of motifs in the P_2_-type Ca^2+^ATPase proteins sequences. Evolutionary bioinformatics was conducted as described in (Zhao et al., 2019). Briefly, candidate protein sequences were selected using the 1,000 Plant Transcriptome (1KP) database (Leebens-Mack et al., 2019).^9^ The amino acid sequences were employed as the query sequences to access the transcriptome data with the criterion of E-value <10^−10^ and coverage >50% by using BLASTP. MAFFT^10^ was applied to align the protein sequences and the phylogenies constructed with the online toolkit RAxML of CIPRES (Miller et al., 2010). The Interactive Tree of Life resource^11^ was used to annotate gene trees.

### Bioinformatic Analysis

The location of the functional domain was to align the identified soybean P_2_-type Ca^2+^ ATPase protein sequence one by one using PHMMER online website.^12^ In addition, the online Gene Structure Display Service (GSDS2.0)^13^ was used to predict the intron structure by comparing the cDNA of the soybean P_2_-type Ca^2+^ ATPase genes with the corresponding genomic DNA sequences.

All the soybean P_2_-type Ca^2+^ ATPase genes were mapped to the chromosomes from the physical location information obtained from the soybean genomic database using Circos (Krzywinski et al., 2009). Multiple collinear scanning toolkit (MCScanX) was used to analyze gene duplication events with the default parameters (Wang et al., 2012). The syntenic analysis maps were constructed using the Dual Synteny Plotter software.^14^

The upstream 2 kb regions from the transcription start site of each soybean P_2_-type Ca^2+^ ATPases genes were extracted from the soybean genome sequence, and used to identify cis-elements by the PlantCARE server.^15^

### Chlorophyll Fluorescence Imaging and Stomatal Aperture Analysis

Chlorophyll fluorescence imaging (Chaerle et al., 2007) was performed using V4 stage soybean seedlings at the control and abiotic stress treatment for 12 h. Whole seedlings were photographed with Manual Plant Explorer™ (PhenoVation, Holland). Each measurement was performed on three replicates for each treatment, with 15 seedlings from each replicate. Stomatal aperture images were taken using Digital Microscope VHX-7000 Series (KEYENCE, Japan) according to (O’Carrigan et al., 2014). Each measurement was performed on three replicates for each treatment, with 50 stomata from each replicate.

### Real-Time Quantitative PCR Analysis of Gene Responses to Abiotic Stresses

Total RNA was isolated from leaves using the EZNA Plant RNA Kit (Omega Bio-Tec, United States) following the manufacturer’s instructions. RNA integrity was verified with 1% agar gel electrophoresis and the RNA concentration was measured using BioDrop uLite (BioDrop, United Kingdom). The first cDNA strand was synthesized from 1 μg of total RNA using Hifair® III 1st Strand cDNA Synthesis Kit with dsDNase (Yeasen, China) according to the manufacturer’s instructions. Real-time quantitative PCR (qPCR) was conducted in Bio-rad CFX96™ (Bio-rad, United States) using Hieff® qPCR SYBR Green Master Mix (None ROX; Yeasen, China). The 15 μl reaction mixture contained 2 μl of a diluted template (10 μl of the generated first-strand cDNA diluted by 90 μl ddH_2_O), 7.5 μl of Hieff® qPCR SYBR Green Master Mix, and 0.4 μl of each of the two gene specific primers (10 μM), 4.7 μl ddH_2_O. The reactions were performed as follows: 95°C for 30 s, 40 cycles at 95°C for 10 s, 60°C for 15 s, and 72°C for 20 s. A melting curve analysis was conducted following each assay to confirm the specificity of the amplicon for each primer pair. Gene-specific primers were designed using Primer 3 (Untergasser et al., 2012). Relative gene expression values were calculated using the 2^−ΔΔ*C*T^ method with the soybean *GmEF1b* as the reference gene (Hu et al., 2009). The gene-specific primers are listed in Supplementary Table S1.

### Data Analysis

Student’s *t*-tests were used to determine significance levels for control and treatment phenotypic data (*F_v_*/*F*_m_ and stomatal aperture). Significance levels: 0.01 < ^*^p ≤ 0.05, 0.001 < ^**^p ≤ 0.01, ^***^p ≤ 0.001. The relative transcript expression levels of each vegetable soybean P_2_-type Ca^2+^ ATPase genes were transformed to log_2_. The data clustering analysis and the quantitative color scheme were performed by Amazing Heatmap.^14^

## Results

### Identification of the P_2_-Type Ca^2+^ ATPase Genes in *Glycine max*

In this study, we first obtained the P_2_-type Ca^2+^ ATPase gene sequences from the *G. max* genome by HmmerWeb version 2.39.0. Twelve P_2_-type calcium ATPase genes from *G. max* were identified by further BLASTp methods of NCBI. We renamed the *Glycine max* P_2B_-type Ca^2+^ ATPase genes (*GmACA1, GmACA2, GmACA8, GmACA9, GmACA10, GmACA11, GmACA12*, and *GmACA13*) and P_2A_-type Ca^2+^ ATPase genes (*GmECA1, GmECA2, GmECA3*, and *GmECA4*) based on close homology to the corresponding Arabidopsis genes (Table 1). The results showed that *GmACAs* and *GmECAs* were distributed on eight soybean chromosomes (Table 1). The lengths of the 12 P_2_-type Ca^2+^ ATPases varied from 1,001 (GmECA3) to 1,105 (GmACA10) residues with an average of 1,053 amino acids. Molecular weights of GmACAs and GmECAs ranged from 109.9 to 120.8 kDa and their theoretical isoelectric points (pIs) ranged from 5.22 (GmECA4) to 8.06 (GmACA8).

### Evolutionary Analysis of the P_2_-Type Ca^2+^ ATPases

**Figure 1:**
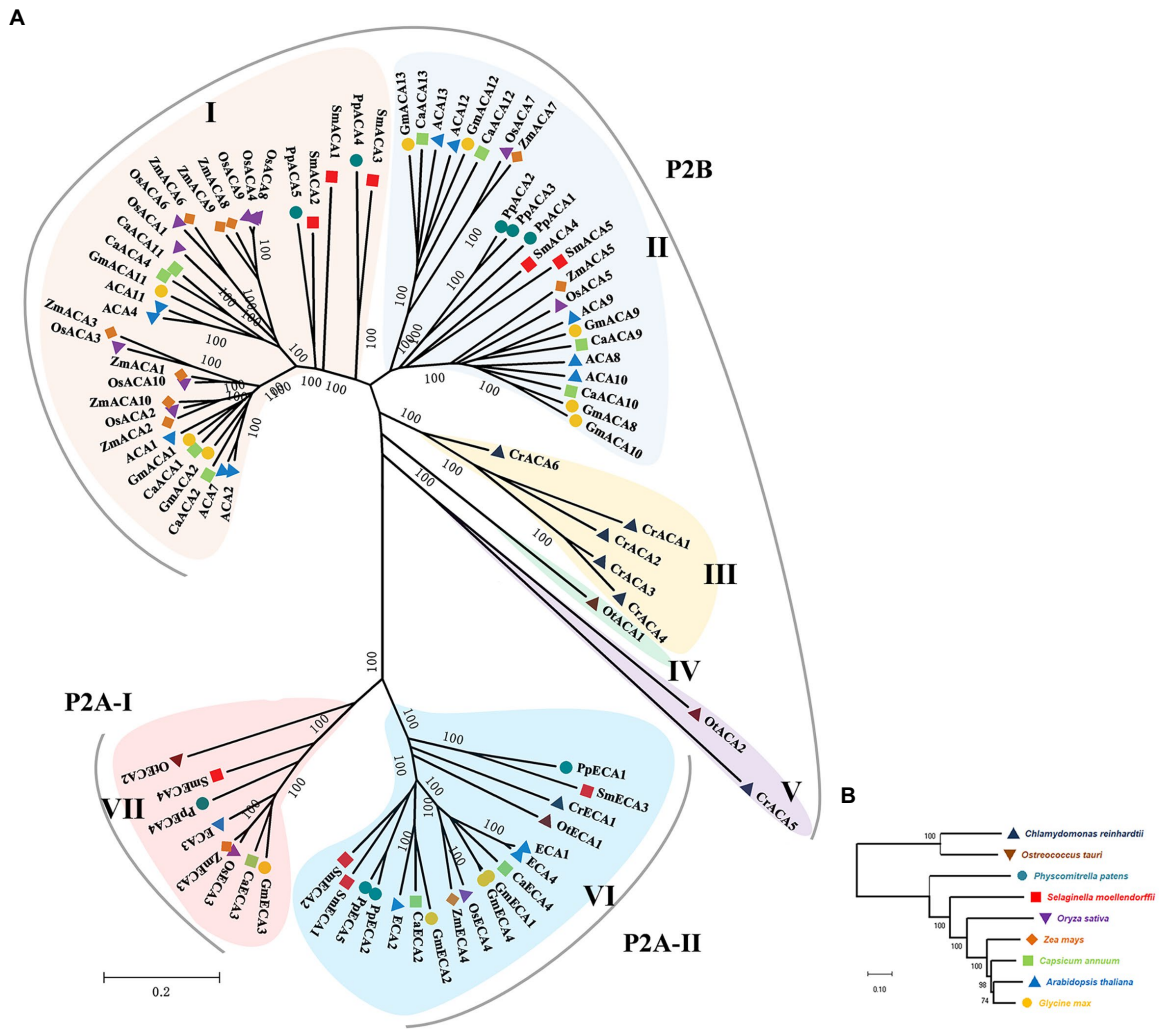
The phylogenetic tree for the P_2_- type ATPases. **(A)** The tree was constructed from a complete alignment of *Chlamydomonas reinhardtii*, *Ostreococcus tauri*, *Physcomitrella patens*, *Selaginella moellendorffii*, *Oryza sativa*, *Zea mays*, *Capsicum annuum*, *Arabidopsis thaliana*, and *Glycine max* P_2_-type ATPases and generated with the MEGA-X program using the Neighbor-Joining method. The resulting groups are shown in different shades of colors. **(B)** Evolutionary relationship of P_2_-type ATPases between green species used to construct phylogenetic trees.

**Figure 2:**
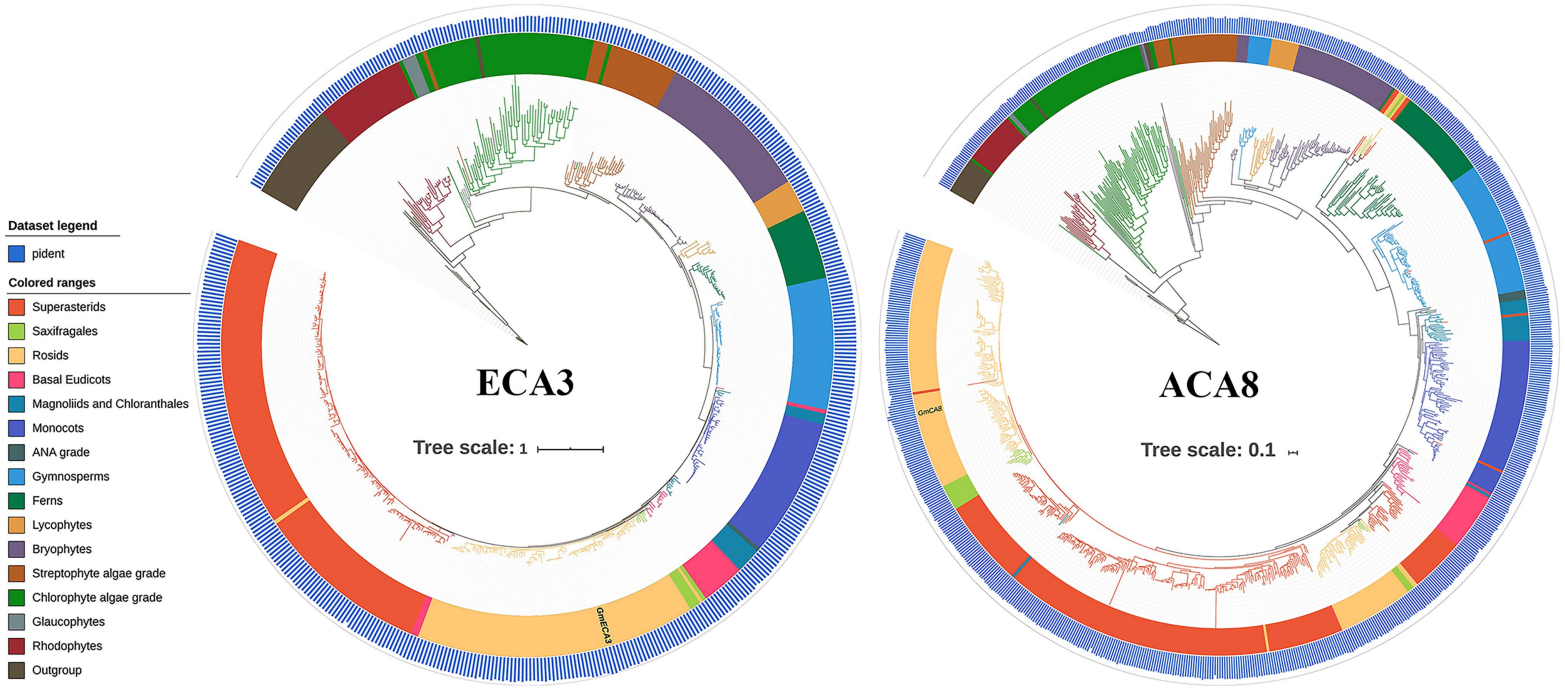
Phylogenetic analysis of GmACA8 and GmECA3. Genes sampled from Chromia algae were used as the outgroup (in the shade of dark brown) and the root of the tree, and the Interactive Tree of Life resource was used to annotate gene trees. The location of GmACA8 and GmECA3 were shown on the phylogenetic trees. The blue bar chart outside the phylogenetic tree represents the percentage of identical matches, which is range from 0 to 100.

To deduce the evolutionary relationship of the P_2_-type Ca^2+^ ATPase genes, a phylogenetic analysis was performed for nine green plant species (Figure 1). Phylogenetic analysis showed that the P_2_-type Ca^2+^ ATPase genes are divided into seven groups. The P_2B_-type Ca^2+^ ATPase genes were assigned to the I–V Groups. Groups I and II contain the most CAs of land plants while Groups III–IV belong to algae (Figure 1). The P_2A_-type Ca^2+^ ATPase proteins were assigned to VI and VII Groups. The GmACAs proteins were most closely related to ACAs in Arabidopsis (Figure 1B). All of the five members (CrCA1, CrCA2, CrCA3, CrCA4, and CrCA6) of *C. reinhardtii* in the order of Chlamydomonadales in Group III were CrCAs. OtCA1 of *O. tauri* in the order of Mamiellales was the only member in Group IV (Figure 1A). The full-length amino acid sequences of GmACA8 and GmECA3 were used to further analyze the evolutionary origin of this family of genes using the 1KP database (Figure 2). We found that the homologs of ECA3s and CA8s were highly conserved in plants and algae with 510 and 820 out of the 1,300 OneKP species. As is presented in Figure 2, the homologs of ECA3s and CA8s have existed in all tested clades of the land plant lineage with high similarity (Bryophyta, Lycophyta, Ferns, Gymnosperm, and angiosperm), as well as the algae (Glaucophyta, Rhodophyta, Chlorophyta, and Streptophytina). This is consisting with the previous study that the P2A ATPases and P2B ATPases are common in both prokaryotes and eukaryotes (Palmgren et al., 2020; Figure 2).

Ten conserved motifs were detected in the P_2_-type Ca^2+^ ATPase protein sequences of all species (Supplementary Figure S1, Supplementary Table S2). Except for CrCA6, 10 motifs were detected in all members of Group I, II, III, and IV. OtCA2 in Group V did not contain motif 5. CrCA5 only contained six motifs without motifs 5, 6, 8, and 9. Motif 7 was missing from all members in Groups VI and VII. Motif 5 was not found in SmECA4 and OtECA2. Overall, P_2_-type Ca^2+^ ATPases in the same evolutionary lineage of green plants had similar motif compositions and arrangements (Supplementary Figure S1).

### Functional Domains in *Glycine max* P_2_-Type Ca^2+^ ATPases

In order to understand the evolutionary trend and potential function of GmACAs and GmECAs, we carried out phylogenetic analysis based on the full-length protein sequences and conserved functional domain analysis of P_2_-type Ca^2+^ ATPases between *G. max* and Arabidopsis (Supplementary Figures S2, S3). P_2_-type Ca^2+^ ATPases from *Glycine max* and Arabidopsis were divided into three evolutionary groups and GmECAs were distributed into two groups. We identified seven conserved domains, containing typical Cation transporter/ATPase, N-terminus, E1-E2 ATPase, Cation transporting ATPase, C-terminus and 8–10 transmembrane, and signal domains. Except for ACA12 and ACA13, all members of Groups I and II contained the N terminal auto-inhibitory domain (Supplementary Figure S2).

### Gene Structure Localization and Synteny Analysis of P_2_-Type Ca^2+^ ATPase Genes

The diversity of genetic structure is one of the mechanisms that promote the evolution of multiple gene families (Garris et al., 2005; Schad et al., 2013). We compared the number and location of exons and introns in the P_2_-type Ca^2+^ ATPase gene sequences of Arabidopsis and *G. max* (Supplementary Figure S4). The results showed that the homologous genes in the same evolutionary groups exhibited similar exon-intron composition. The number of exons in *GmACAs* and *GmECAs* varied greatly from 1 to 42. Members in Group II of P_2_-type Ca^2+^ ATPase genes contained the largest and least number of exons, *GmACA10* contained the most 42 exons, *GmACA8* contained 38 exons, while *GmACA12* and *GmACA13* only contained 1 exon. In Group VII and VI, *GmECA3* contained 35 exons, *GmECA2* contained nine exons, *GmECA1 and GmECA4* contained eight exons (Supplementary Figure S4).

**Figure 3:**
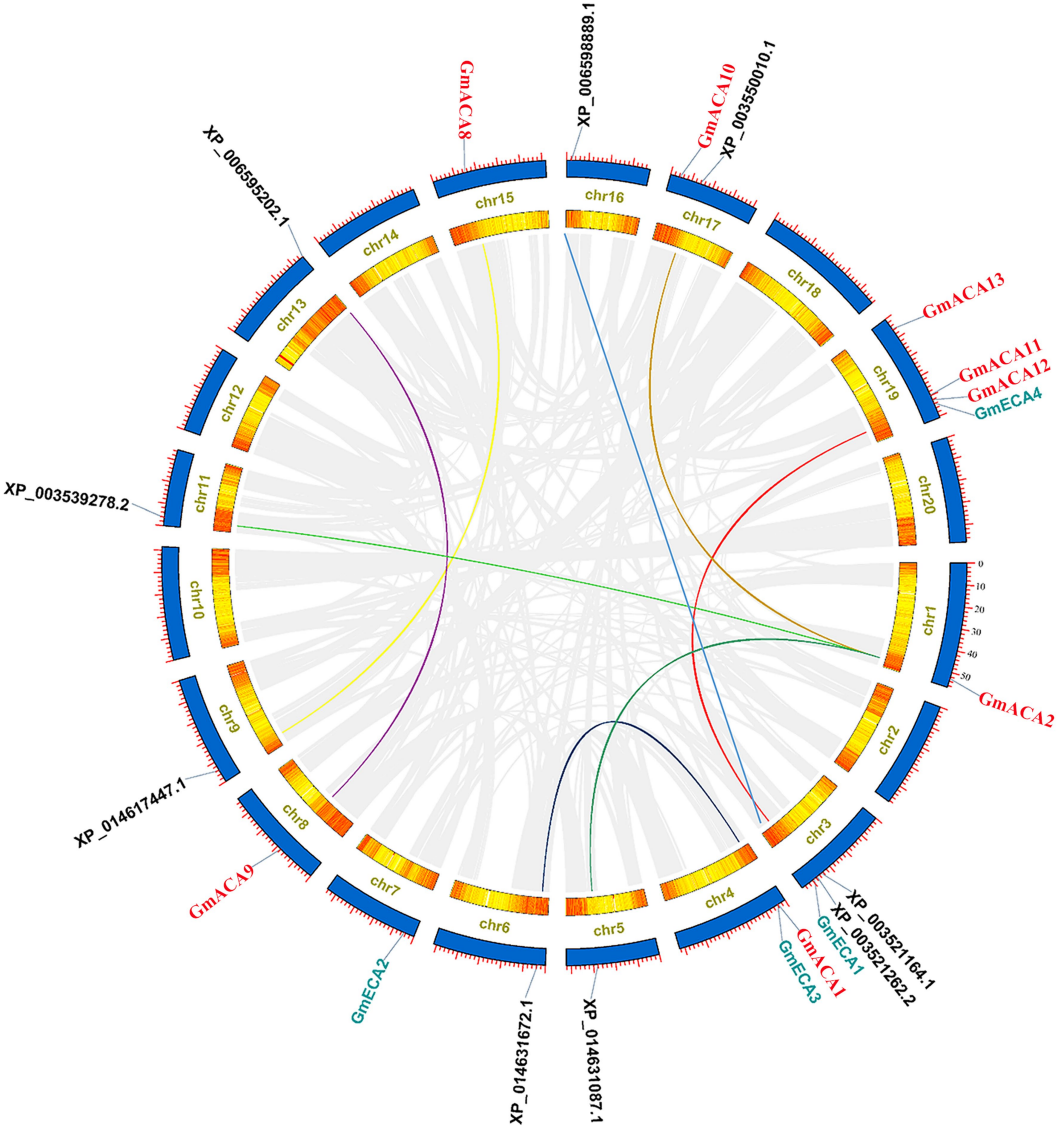
Chromosome localization and collinearity analysis of *Glycine max* P_2_-type ATPases family proteins. Colored lines indicate P_2_-type ATPases syntenic blocks in the soybean genome. Gray lines indicate all syntenic blocks except P_2_-type ATPases. The shades of color in the inner chromosome chromosomes represent the number of genes at the location of the chromosome, and yellow to red indicates the number of genes from low to high.

We then analyzed the gene duplication of *GmACAs* and *GmECAs* in the *G. max* genome. A total of 12 P_2_-type Ca^2+^ ATPase genes were unevenly distributed in eight chromosomes. Chromosome (Chr) 19 had the most genes, including *GmACA11*, *GmACA12*, *GmACA13*, and *GmECA4*. There was no tandem duplication in the *GmACAs* and *GmECAs*, but there were nine pairs of segmental duplicates. For instance, *GmACA2* has three homologous genes on chromosomes 5, 11, and 17, respectively (Figure 3; Supplementary Figure S5).

### Cis–Elements Analysis of the Promoters of *Glycine max* P_2_-Type Ca^2+^ ATPase Genes

**Figure 4:**
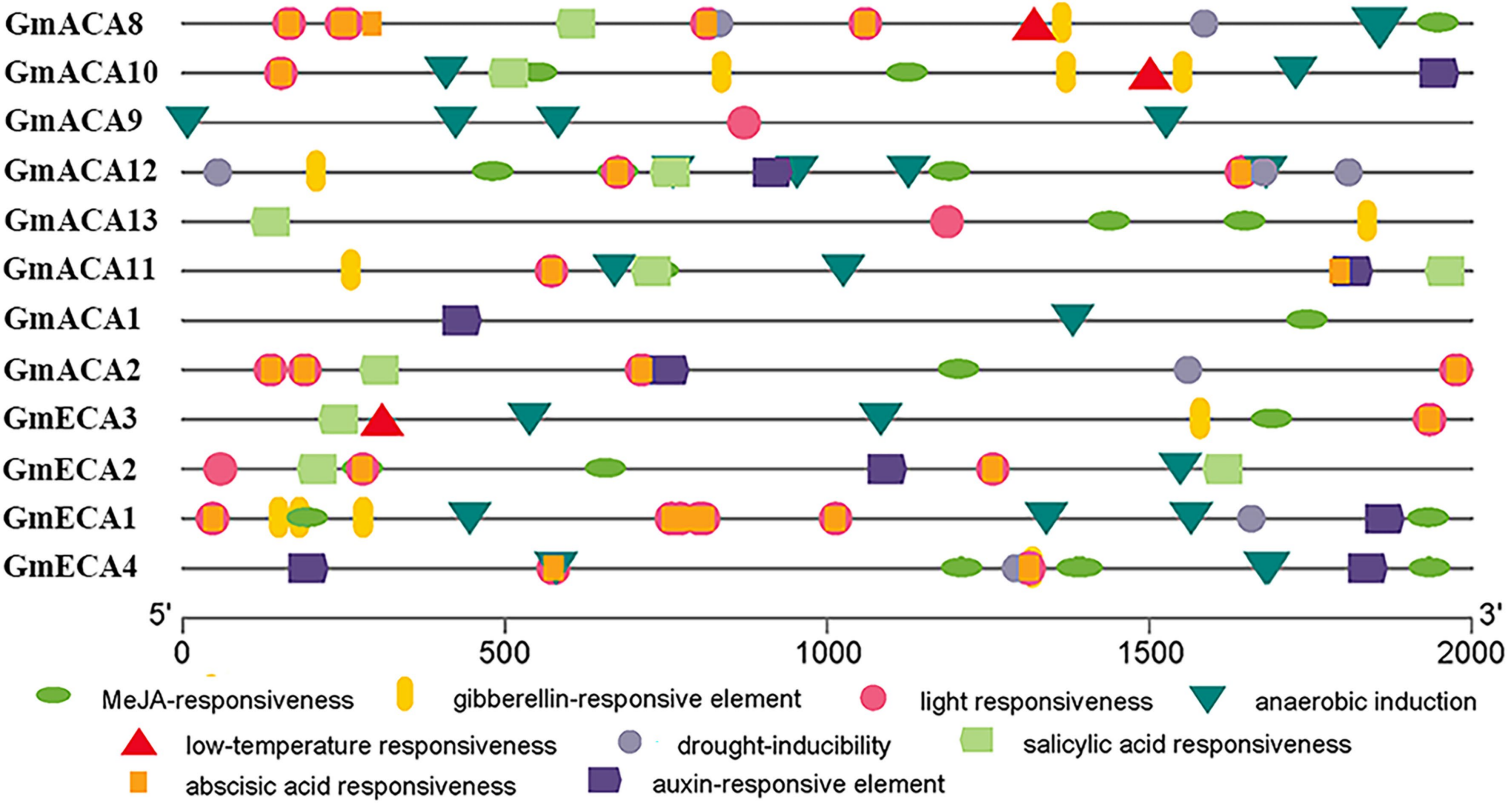
Analysis of the cis-elements of the 2,000 bp promoter sequence upstream of the initial codon of *Glycine max* P_2_-type ATPases genes from the evolutionary level. Different color graphics represent different cis-elements and positions on the promoter.

Different cis-elements in gene promoters determine gene spatial and temporal-specific expression (Wang et al., 2013). Thus, we analyzed the cis-elements in the promoter region of *GmACAs* and *GmECAs* (Figure 4). The results showed that the cis-elements in the promoters of *GmACAs* and *GmECAs* are mainly divided into two types. Type I was relevant to phytohormones, including MeJA, gibberellin, salicylic acid, abscisic acid, and auxin responsiveness cis-elements. Type II was related to abiotic stresses, including light, anaerobic, low-temperature, and drought responsiveness cis-elements (Figure 4).

### Chlorophyll Fluorescence Imaging Assay of Soybean Under Abiotic Stresses

**Figure 5:**
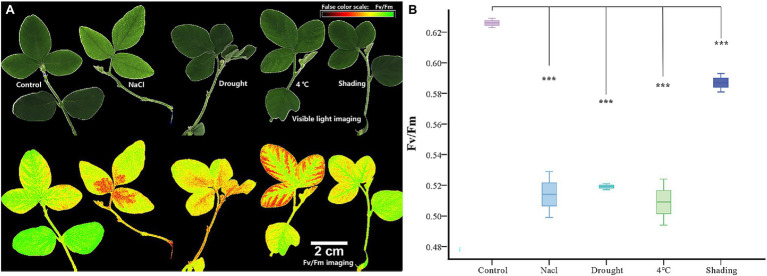
Chlorophyll fluorescence imaging and chlorophyll fluorescence yield (*F_v_*/*F_m_*) index in vegetable soybean under control and abiotic stress. **(A)** Abiotic stress damage in soybean leaves is visualized by chlorophyll fluorescence imaging. The panels show false-color images of the chlorophyll fluorescence yield (*F_v_*/*F_m_*) of leaves after 12 h stress. **(B)** Quantitative comparison of abiotic stress damage to soybean leaves determined by chlorophyll fluorescence imaging (*F_v_*/*F_m_*). ***p ≤ 0.001.

We then conducted experiments to validate these predictions for GmACAs and GmECAs in soybean. False color images of chlorophyll fluorescence yield (*F_v_*/*F_m_*) from leaves indicated a significant difference in damage to soybean plants under drought, cold, salt, and shading stresses (Figure 5). The average *F_v_*/*F_m_* of leaves was significantly reduced by drought, salt, and cold stresses, implicating a serious impact on the overall photosynthetic capacity of leaves. However, less damage was observed in leaves as indicated by *F_v_*/*F_m_* values after shading treatment.

### Expression Patterns of *GmACAs* and *GmECAs* in Response to Abiotic Stress

**Figure 6:**
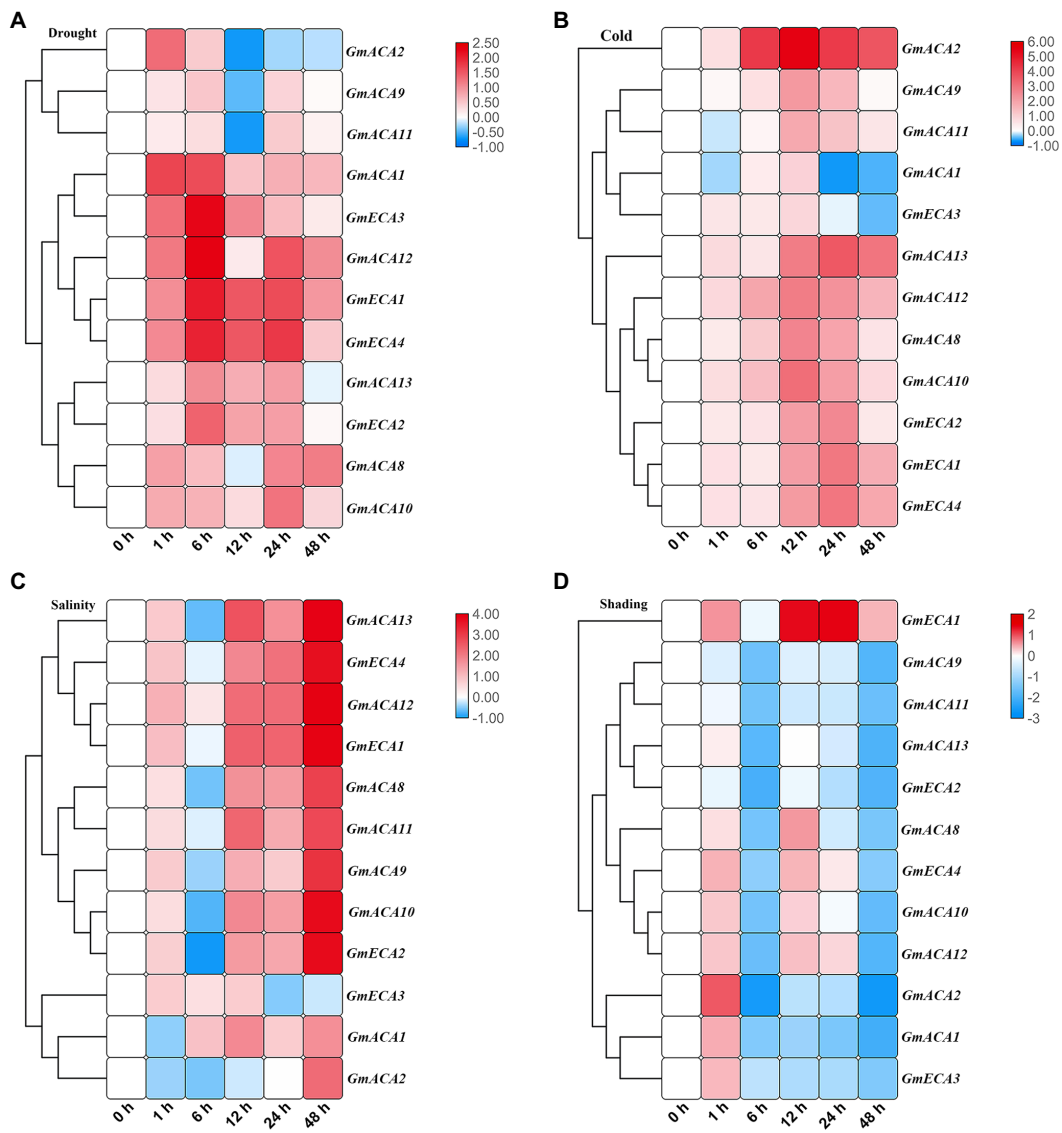
Heat map expression profiles of P_2_-type ATPases genes in leaf under four different abiotic stresses. **(A)** drought stress; **(B)** cold (4°C); **(C)** salinity stress (250 mM); **(D)** shading stress. The color bar in each panel represents log_2_ expression values, blue indicates lower and red higher transcript abundance compared to the relevant control. The annotated color quantitation scale is seen as a vertical column on the right side of the heat map. Abiotic stress stages used for expression profiling are mentioned at the bottom of each column.

The expression of all 12 P_2_-type Ca^2+^ ATPase genes was analyzed under different abiotic stresses to further validate their potential functions (Figure 6). Overall, drought, salt, and cold stress caused more serious damage to vegetable soybean, where the expression of *GmACAs* and *GmECAs* were largely up-regulated. Meanwhile, less damage was found under shading stress and P_2_-type Ca^2+^ ATPase genes were mainly down-regulated. Most of *GmACAs* and *GmECAs* were up-regulated during the five time points under drought stress, except for *GmACA2* (down-regulated in 12, 24, and 48 h), as well as *GmACA8*, *GmACA9*, and *GmACA11* (down-regulated in 12 h; Figure 6A). Similar results were found under the cold treatment (Figure 6B). Only the expression of *GmACA1*, *GmECA3*, and *GmACA11* decreased compared with the control. Interestingly, *GmACA2* showed a significantly high expression from 6 to 48 h of cold stress as compared to other *GmACAs* and *GmECAs*. Under salt stress, most of *GmACAs* and *GmECAs* showed an increase throughout the treatment. There was a sudden drop in the expression of *GmACA8*, *GmACA9*, *GmACA10*, *GmACA11*, *GmACA13*, and *GmECA2* at 6 h of salt treatment. The expression level of *GmECA3* reached peaks at 12 h followed by a decrease at 24 and 48 h (Figure 6C). Moreover, the expression pattern of *GmACAs* and *GmECAs* under shading stress was dramatically different in contrast to drought, salinity, and cold treatments. There was a short-term up-regulation in the expression level of all 12 *GmACAs* and *GmECAs* under 1-h shading stress (Figure 6D). Then, almost all of the genes showed down-regulation except for *GmECA1*, which kept higher expression compared to the control.

### Stomatal Aperture Assay of Soybean Under Abiotic Stresses

**Figure 7:**
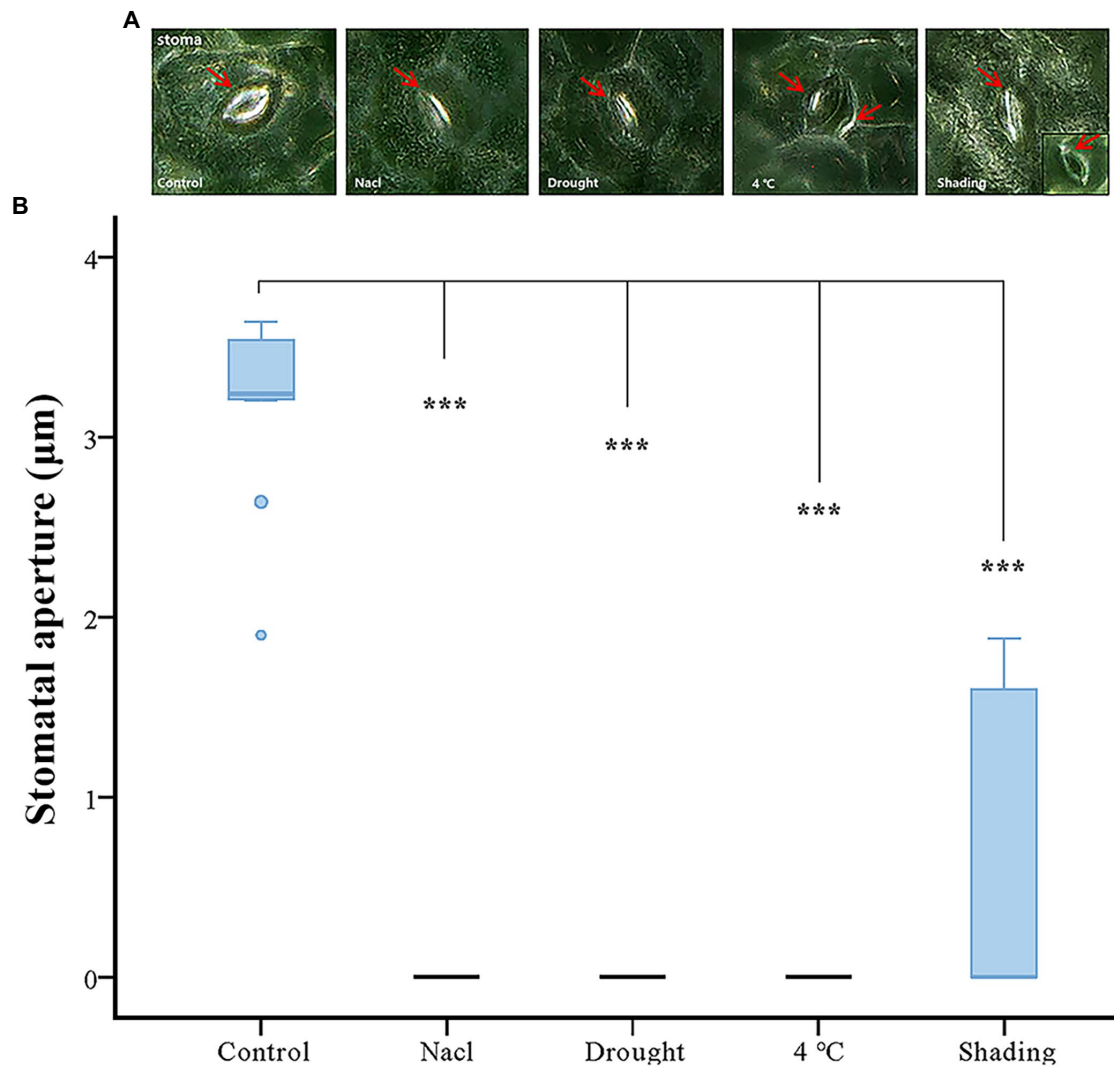
Effect of abiotic stress on stomatal aperture. **(A)** The microscope of stomatal movement of vegetable soybean leaves under control and abiotic stress. The red arrow indicates the position of the stoma. **(B)** Inhibition of stomatal aperture by abiotic stress. ***p ≤ 0.001.

Taking into consideration that the function of GmACAs and GmECAs in regulating [Ca^2+^]_cyt_ are closely related to the stomatal aperture. We performed an imaging analysis of the stomatal state under different stresses. The stomatal opening degree was quantified using the ImageJ program (Figure 7A). The results showed that the stomatal opening of soybean was significantly reduced by the drought, cold and salt stresses to almost complete closure (Figure 7B). However, the stomata displayed less degree of closure in response to the shading treatment. We further conducted a correlation analysis between the expression levels of Ca^2+^ ATPase genes and the stomatal aperture under four abiotic stresses, and found that the expression of Ca^2+^ ATPase genes was negatively correlated with the stomatal aperture (Supplementary Figure S6).

## Discussion

### The Evolution of P_2_-Type Ca^2+^ ATPases Is Linked to Stress Tolerance in Land Plants

P_2_-type Ca^2+^ ATPases are involved in maintaining the accurate concentration of Ca^2+^, Mn^2+^, and Zn^2+^ in the cytosol located in different membranes (Feng et al., 2002; Li et al., 2008). With the completion of many plant genome assemblies, it is convenient to compare the characteristics of gene families to understand the evolutionary relationships among different species. Previous studies showed that there were 14 P_2_-type Ca^2+^ ATPase genes in *A. thaliana* (Rahmati Ishka, 2015), 29 in *Glycine soja* (Sun et al., 2016), 11 in rice (Li et al., 2015), 13 in wheat (Aslam et al., 2017), 4 in *O. tauri*, 7 in *C. reinhardtii*, 11 in *P. patens*, and 9 in *S. moellendorffii* (Pedersen et al., 2012). In this study, 12 P_2_-type ATPase genes were firstly identified from the *G. max* genome and the evolutionary origin, the expression level of *GmACAs* and *GmECAs* and their potential function on the stomatal movement of vegetable soybean leaves under abiotic stress were characterized.

P_2_-type Ca^2+^ ATPases system is responsible for the extrusion of cytosol Ca^2+^. Its high affinity and low turnover efflux ability could make the cytosol Ca^2+^ concentration back to a few micromolar after signal mediated influx (Kudla et al., 2010). In the process of plant evolution, different members of the P_2_-type Ca^2+^ ATPase family seemed to have their unique spatial positioning and functions. In Arabidopsis, It was reported that AtACA1, AtACA2, and AtECA1 are localized at the endoplasmic reticulum; AtACA8, AtACA9, AtACA10, and AtACA12 are localized at the plasma membrane; AtACA11 is localized at the vacuolar membrane; AtECA3 was localized at the Golgi (Table 1; Liang and Sze, 1998; Bonza et al., 2000; Hwang et al., 2000; Schiøtt et al., 2004; Lee et al., 2007; George et al., 2008; Li et al., 2008; Limonta et al., 2014; Rahmati Ishka, 2015). *Ostreococcus Tauri* is a unicellular alga, which does not have the spatial ability to contain bigger organizational structures (Raven, 2002), indicating less requirement for Ca^2+^ transport. Therefore, the regulation of calcium signals in *Ostreococcus Tauri* cells is relatively simple compared with the vascular plants such as Arabidopsis and soybean. Phylogenetic tree analysis found that *Ostreococcus Tauri* contained only 4 P_2_-type Ca^2+^ ATPase members. From the evolutionary origin of GmACA8 and GmECA3 (Figure 2), it was found that the genetic relationship of P_2_-type Ca^2+^ ATPases was farther from that of algae, and the number of species distributed by GmECA3 (103 species) in algae is less than the number of species distributed by GmACA8 (129 species). The results suggested that there are differences between P_2_-type Ca^2+^ ATPase genes in algae and vascular plants, which might be a sign of functional differentiation in calcium signal regulation and stress tolerance.

### GmACAs and GmECAs Are Key Genes in Abiotic Stress Response in Soybean

Calcium signals were reported to be involved in abiotic stress response and induced stomatal closure. Drought stress, abscisic acid (ABA), hydrogen peroxide, cold, the elevation of external Ca^2+^, and atmospheric CO_2_ all induce stomatal closure. [Ca^2+^]_cyt_ signals could be generated by influx and efflux of the ion from the extracellular space, or by storage and release from intracellular compartments, such as the endoplasmic reticulum (ER), Golgi, plastid, and vacuole. By recruiting different stores, distinct spatial patterns of [Ca^2+^]_cyt_ could be generated (Radin, 1984; Allen et al., 2001; Lim et al., 2015; Tombesi et al., 2015). Ca^2+^ pumps (CAs and ECAs) are responsible for the release of [Ca^2+^]_cyt_ to the extracellular space and the influx to the intracellular Ca^2+^ stores. In rice, *OsCAs* family genes were regulated by four different osmotic-related abiotic stresses (PEG, NaCl, drain, and ABA; Li et al., 2015). In wheat, *ECAs* induced up-regulation with the increase of Ca^2+^ concentration. We analyzed, for the first time, the expression patterns of P_2_-type Ca^2+^ ATPase family genes in soybean under four abiotic stresses (drought, cold, salt, and shading; Figure 6). The results showed that the expression levels of *GmACAs* and *GmECAs* were regulated by abiotic stress (Figure 6). Correlation analysis between the stomatal aperture and the expression levels of Ca^2+^ ATPase genes under four abiotic stresses showed that soybean Ca^2+^ ATPase genes were negatively correlated with the stomatal aperture, indicating that the up-regulated expression of Ca^2+^ ATPase genes may regulate stomatal closure (Supplementary Figure S6). Further research found that all drought, cold, salt, and shading stress leads to stomatal closure (Figure 7), which is consistent with previous studies (Gibbons and Smillie, 1980; Pieruschka et al., 2006; Wang et al., 2015). We speculated that different subcellular localization of P_2_-type Ca^2+^ ATPases on intracellular membranes might be involved in regulating Ca^2+^ signal under different abiotic stresses. Therefore, it is of great significance to study the expression pattern of P_2_-type Ca^2+^ ATPase genes under abiotic stress for its potential function in regulating stomatal movement (Figures 6, 7).

**Figure 8:**
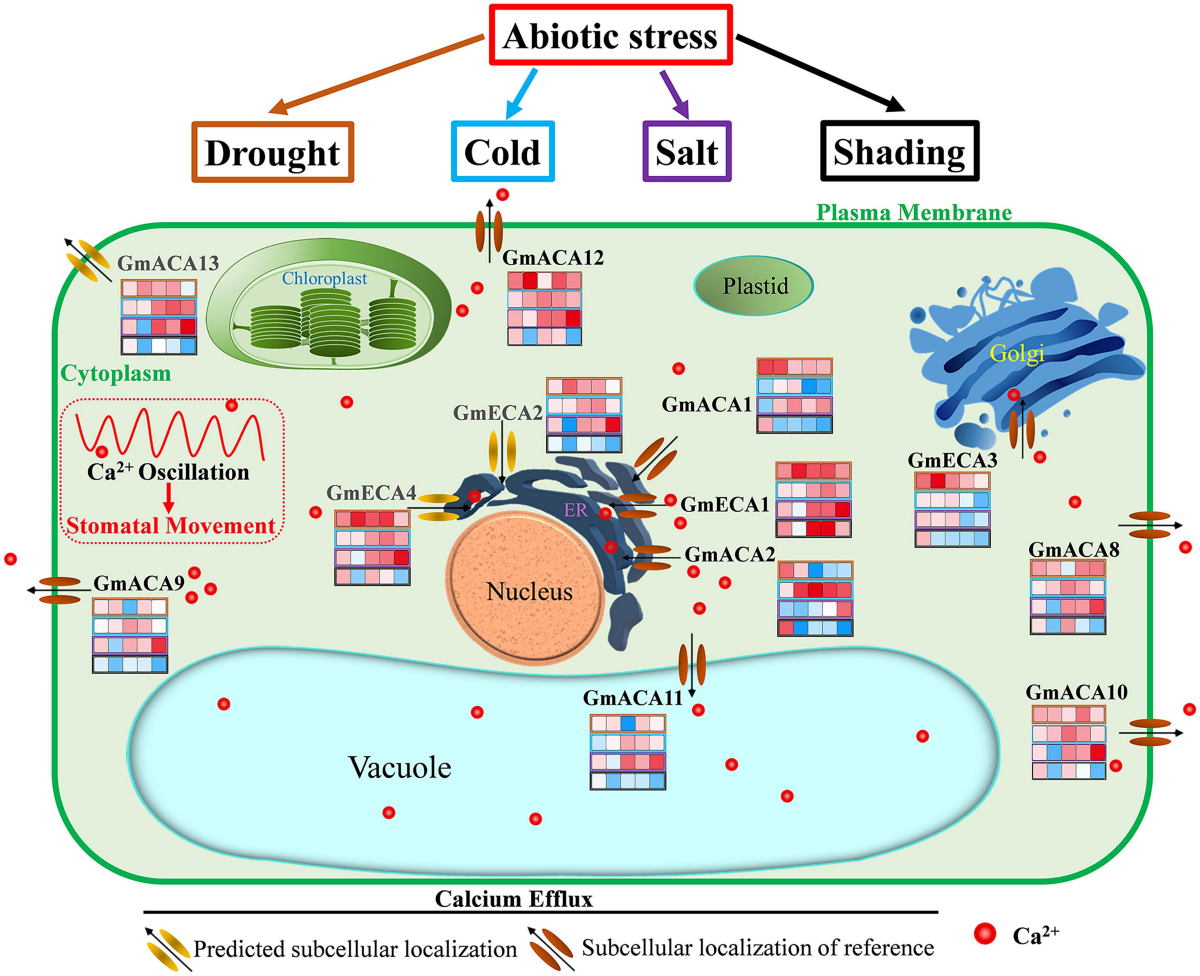
Overview of the regulation of calcium efflux by P_2_-type Ca^2+^ pump (GmACAs and GmECAs) in vegetable soybean cells. By referring to the identified transport system of the Ca^2+^ pump in Arabidopsis, the schematic representation of the P_2_-type Ca^2+^ pump transport system in soybean was summarized. Grey colors of gene names represent predicted subcellular localizations. The heat map represents the expression level of each gene at 1, 6, 12, 24, and 48 h after drought, cold, salt, and shading stress (refer to Figure 6). See the text and Table 1 for discussion and references.

Based on our research and previous studies, we summarized the transport regulation system of the Ca^2+^ pump in vegetable soybean cells (Figure 8). The 12 P_2_-type Ca^2+^ATPase family genes were divided into four types according to their subcellular localization. The four types of GmACAs and GmECAs are located in the plasma membrane (GmACA8, GmACA9, GmACA10, GmACA12, and GmACA13), endoplasmic reticulum (GmACA1, GmACA2, GmECA1, GmECA2, and GmECA4), tonoplast (GmACA11), and Golgi (GmECA3) intracellular membrane systems, respectively. According to the temporal and spatial expression of GmACAs and GmECAs under different stresses, all the genes located on the plasma membrane (*GmACA8*, *GmACA9*, *GmACA10*, *GmACA12*, and *GmACA13*) and the tonoplast (*GmACA11*) were up-regulated by drought, cold, and salt stress, and down-regulated by shading stress. Among the genes located at the endoplasmic reticulum, only *GmECA2* and *GmECA4* showed similar expression profiles. The results revealed that P_2_-type Ca^2+^ ATPase genes located at the endoplasmic reticulum (GmACA1, GmACA2, GmECA1, GmECA2, and GmECA4) might produce functional differentiation in response to different abiotic stresses. It has also shown that when plants are severely stressed, Ca^2+^ pumps located on the plasma membrane and tonoplast are preferentially mobilized in a unified manner, which can rapidly generate Ca^2+^ oscillation to regulate plant responses to external stresses, such as stomata closure. Moreover, *GmACA1* might be involved in the negative regulation of cold and shading stress. *GmECA1* was up-regulated under four abiotic stresses, especially under shading stress, which might positively regulate stomatal closure under shading stress. SCA1, the homologous gene of *GmACA2* in this study, was up-regulated after salt stress (Aslam et al., 2017). Expression patterns of the most homologous genes *GmACA2* under salt stress are consistent with that of *AtACA2* and *AtACA4* (Geisler et al., 2000a; Anil et al., 2007). It showed that *GmACA2* could positively regulate cold and salt stress. The expression level of *AtACA8* was found to be up-regulated under cold stress which was consistent with the results of a previous study (Schiøtt and Palmgren, 2005). *GmECA3* may play a negative regulatory role under cold, salt, and shading stress. *OsACA6* transcript levels were enhanced in response to salt, drought, abscisic acid and heat, and overexpression of *OsACA6* in tobacco can improve salt, drought, and cold tolerance of transgenic lines (Huda et al., 2013a, 2014). It is suggested that the *OsCA6* homologous gene *GmACA11* may regulate plant stress resistance by regulating stomatal closure.

## Conclusion

This study was the first genome-wide analysis of the P_2_-type Ca^2+^ ATPase family in *G. max* responsive to different abiotic stresses (drought, cold, salt and shading stress). The effect of the Ca^2+^ pump on stomatal opening under different stress was speculated through the spatial location and temporal expression of the P_2_-type Ca^2+^ ATPase family genes in soybean. *GmACA8, GmACA9, GmACA10, GmACA12, GmACA13*, and *GmACA11* might promote stomatal closure under drought, cold and salt stress; *GmECA1* might regulate stomatal closure in shading stress. *GmACA1* and *GmECA3* might have a negative function on cold stress. The roles of *GmACAs* in regulating tolerance to different stress should be further evaluated through genetic transformation and functional analysis in soybean and other model organisms such as Arabidopsis, yeast, and *Xenopus* oocytes.

### Significance Statement

The Ca^2+^ pump belongs to the P2-type calcium ATPases gene family, which is responsible for cellular Ca^2+^ transport and plays an important role in plant development and response to biotic and abiotic stresses. The results laid an important foundation for further study on the function of P2-type calcium ATPases genes GmACAs and GmECAs for soybean abiotic-resistant breeding.

## Data Availability Statement

The datasets presented in this study can be found in online repositories. The names of the repository/repositories and accession number(s) can be found in the article/Supplementary Material.

## Author Contributions

SX, JW, XF, and GC conceived and designed the experiments. JW, XF, SZ, SL, TS, and YZ performed the experiments. JW, GC, Z-HC, and SX analyzed the data and wrote the paper. JW, YZ, and FX contributed to reagents/materials/analysis tools. All authors contributed to the article and approved the submitted version.

## Funding

The research was supported by grants from the Key R&D Program of Zhejiang Province (2021C02009) and National Key R&D Program of China (2018YFD0100901).

## Conflict of Interest

The authors declare that the research was conducted in the absence of any commercial or financial relationships that could be construed as a potential conflict of interest.

## Publisher’s Note

All claims expressed in this article are solely those of the authors and do not necessarily represent those of their affiliated organizations, or those of the publisher, the editors and the reviewers. Any product that may be evaluated in this article, or claim that may be made by its manufacturer, is not guaranteed or endorsed by the publisher.

## Footnotes

^1^ https://www.ncbi.nlm.nih.gov

^2^ http://pfam.xfam.org/

^3^ https://www.ebi.ac.uk/Tools/hmmer/search/hmmsearch

^4^ https://blast.ncbi.nlm.nih.gov

^5^ http://web.expasy.org/compute_pi/

^6^ http://web.expasy.org/protparam/

^7^ http://linux1.softberry.com

^8^ http://meme-suite.org/tools/meme

^9^ https://sites.google.com/a/ualberta.ca/onekp/

^10^ https://mafft.cbrc.jp/alignment/software/

^11^ https://itol.embl.de/

^12^ https://www.ebi.ac.uk/Tools/hmmer/search/phmmer

^13^ http://gsds.cbi.pku.edu.cn

^14^ https://github.com/CJ-Chen/TBtools

^15^ http://bioinformatics.psb.ugent.be/webtools/plantcare/html/

## Supplementary Material

Supplementary Figure S1 Motif compositions of the P2-type ATPases from nine different plant species. Inner layer: An unrooted phylogenetic tree constructed using MEGA-X with the neighbor-joining method. The resulting groups are shown in different shades of colors. P2-type ATPases in soybeans are shown with a highlighted background. Outer layer: Distribution of the conserved motifs in P2-type ATPases proteins. The differently colored boxes represent different motifs and their positions in each P2-type ATPases protein sequence.

Supplementary Figure S2 Functional domains analysis of P2-type ATPases proteins in *Arabidopsis* and *Glycine max* from an evolutionary level. The differently colored boxes represent different conserved domains and their positions in each P2-type ATPases protein sequence.

Supplementary Figure S3 Alignment of multiple soybean P2-type ATPases proteins and conserved domains amino acid sequences.

Supplementary Figure S4 Gene structures composition of the P2-type ATPases genes from *Arabidopsis* and *Glycine max*. Exon-intron structures of P2-type ATPases genes. Solid green boxes indicate untranslated 5′- and 3′-regions; solid yellow boxes indicate exons; and black lines indicate introns. The number indicates the phases of the corresponding introns. The protein length can be estimated using the different scales at the bottom.

Supplementary Figure S5 Schematic representations of the chromosomal distribution of the soybean P2-type ATPases genes. The chromosome number is indicated to the left of each chromosome.

Supplementary Figure S6 Correlation analysis of expression levels and the stomatal aperture of P2-type ATPases genes under four abiotic stresses.
