# Supplementary figures and images for: Evolutionary and Regulatory Pattern Analysis of Soybean Ca2+ ATPases for Abiotic Stress Tolerance
Source: Front Plant Sci. 2022 May 19;13:898256. doi: 10.3389/fpls.2022.898256 (PMC9161174; doi:10.3389/fpls.2022.898256)

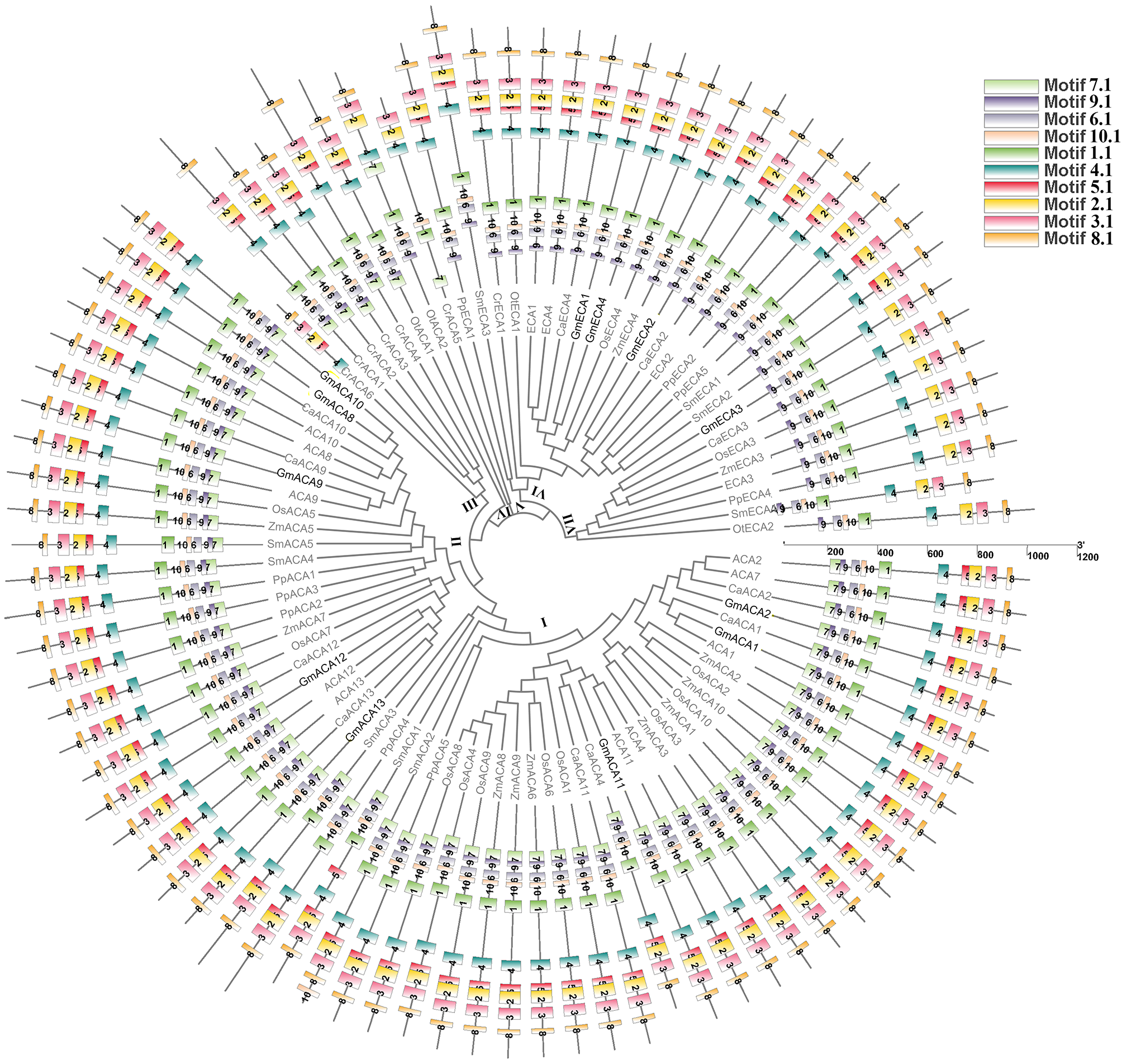

Supplement: Supplementary Figure S1 — Motif compositions of the P2-type ATPases from nine different plant species. Inner layer: An unrooted phylogenetic tree constructed using MEGA-X with the neighbor-joining method. The resulting groups are shown in different shades of colors. P2-type ATPases in soybeans are shown with a highlighted background. Outer layer: Distribution of the conserved motifs in P2-type ATPases proteins. The differently colored boxes represent different motifs and their positions in each P2-type ATPases protein sequence. [file Image_1.TIF]

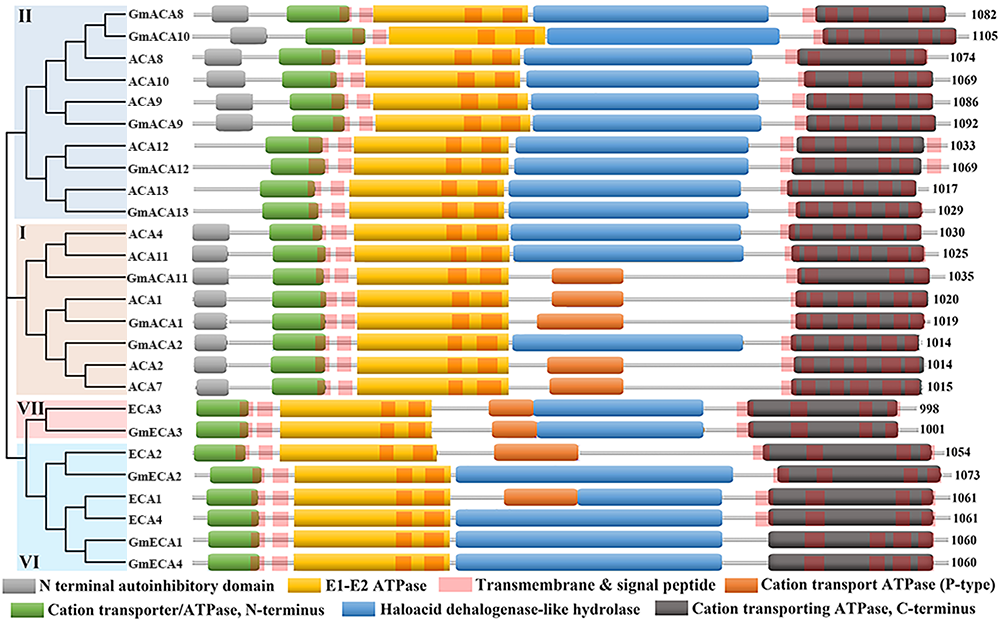

Supplement: Supplementary Figure S2 — Functional domains analysis of P2-type ATPases proteins in Arabidopsis and Glycine max from an evolutionary level. The differently colored boxes represent different conserved domains and their positions in each P2-type ATPases protein sequence. [file Image_2.TIF]

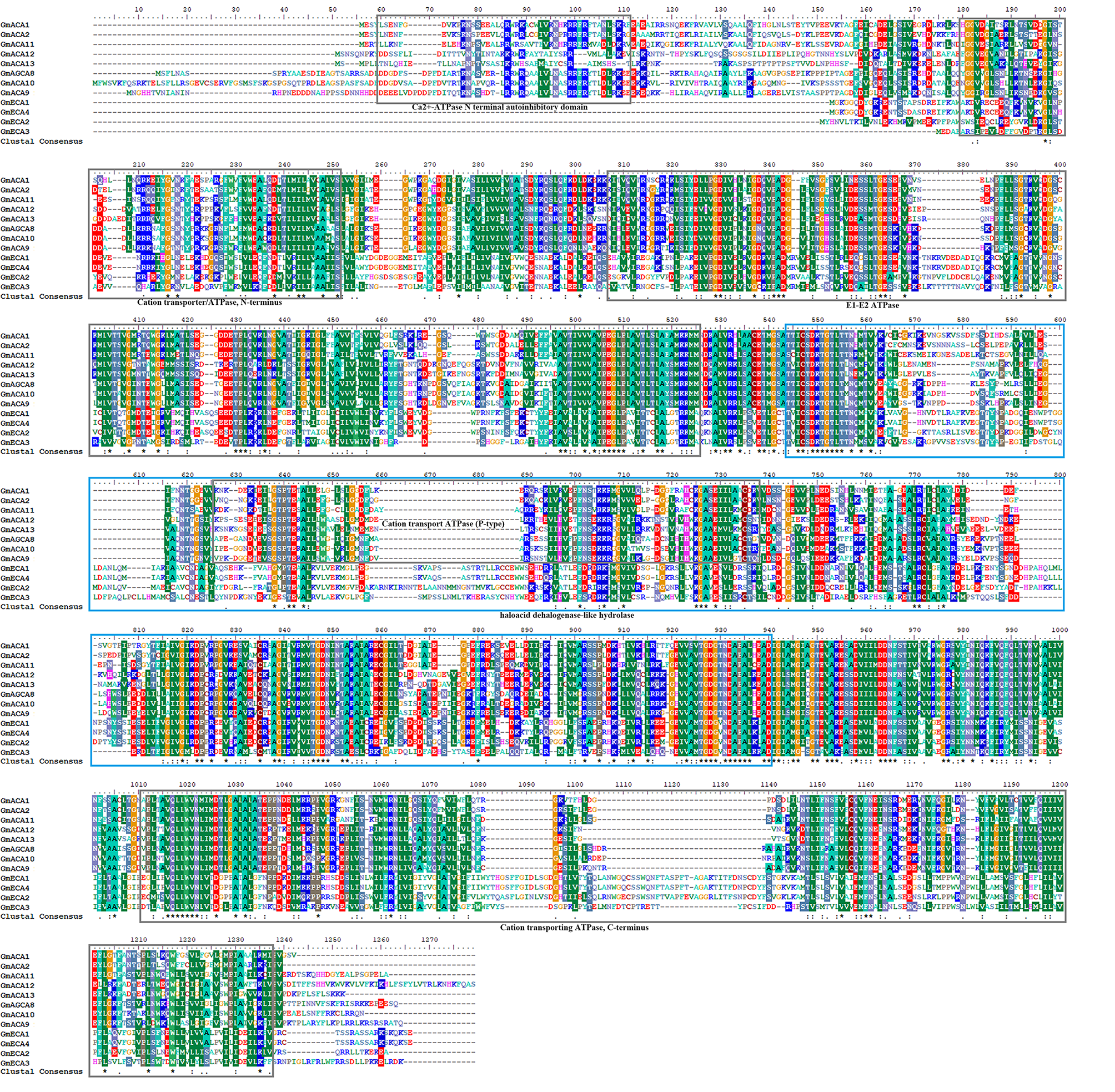

Supplement: Supplementary Figure S3 — Alignment of multiple soybean P2-type ATPases proteins and conserved domains amino acid sequences. [file Image_3.TIF]

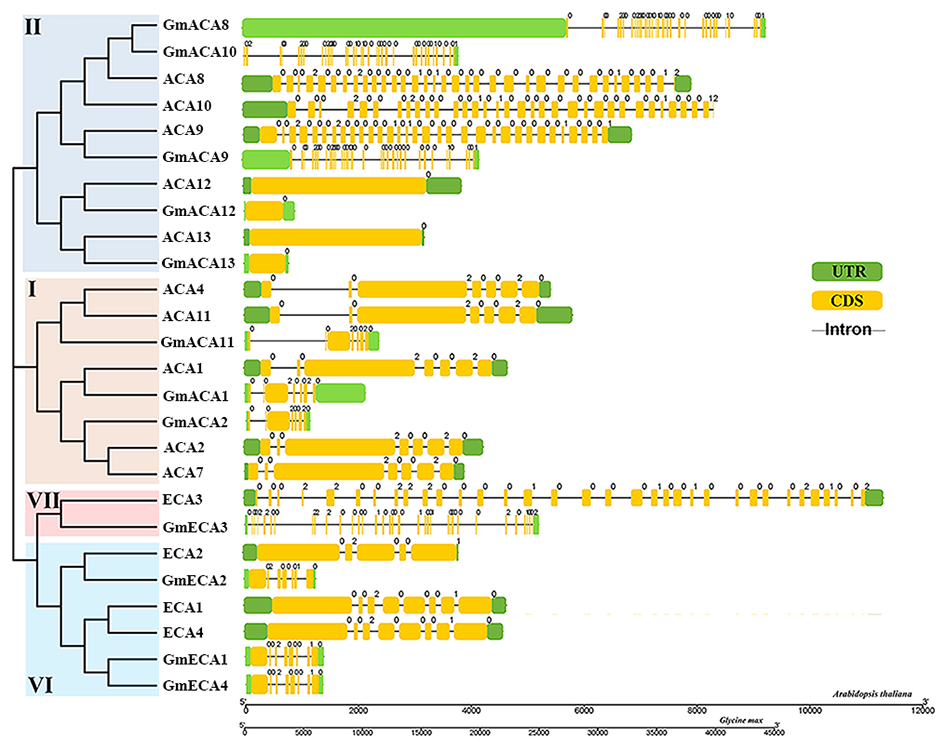

Supplement: Supplementary Figure S4 — Gene structures composition of the P2-type ATPases genes from Arabidopsis and Glycine max. Exon-intron structures of P2-type ATPases genes. Solid green boxes indicate untranslated 5′- and 3′-regions; solid yellow boxes indicate exons; and black lines indicate introns. The number indicates the phases of the corresponding introns. The protein length can be estimated using the different scales at the bottom. [file Image_4.TIF]

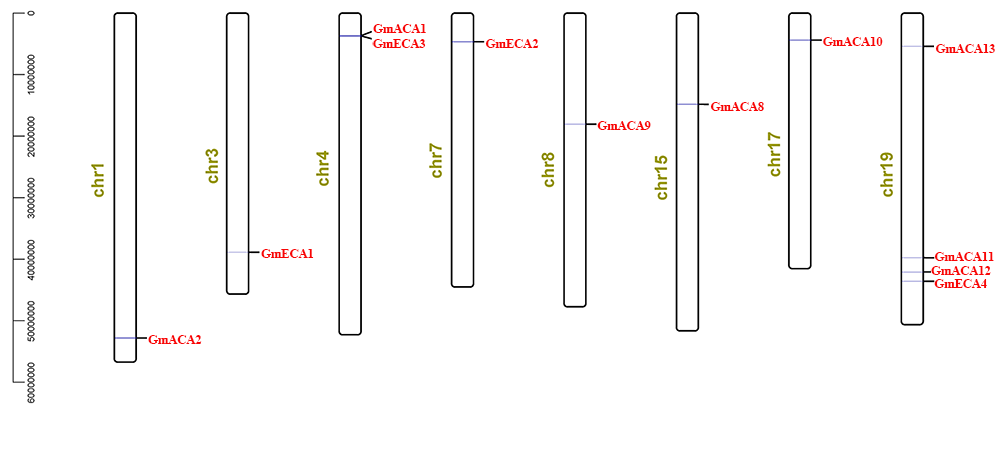

Supplement: Supplementary Figure S5 — Schematic representations of the chromosomal distribution of the soybean P2-type ATPases genes. The chromosome number is indicated to the left of each chromosome. [file Image_5.TIF]

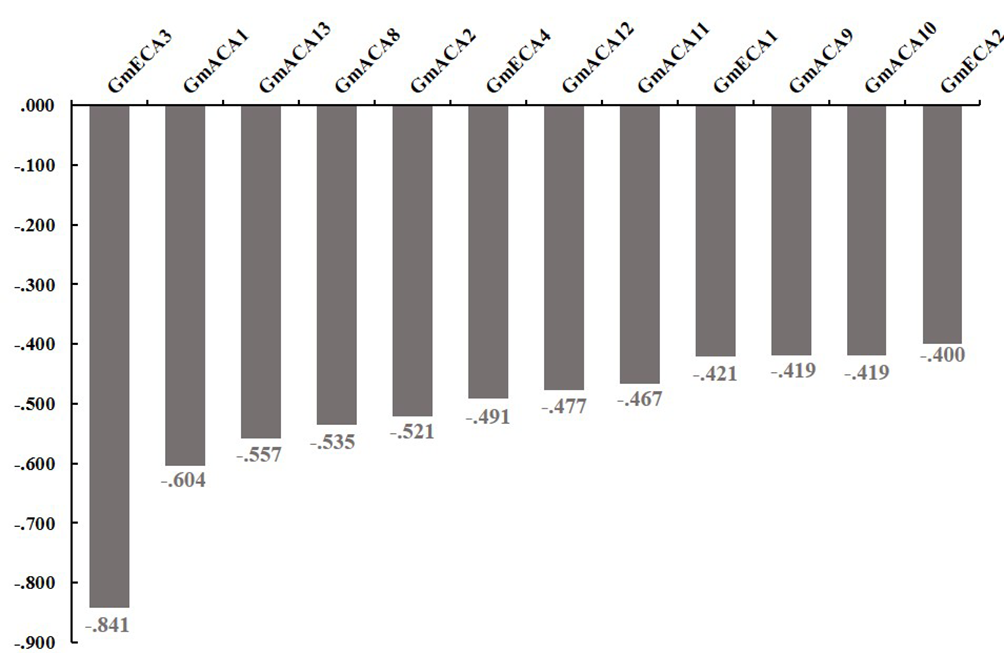

Supplement: Supplementary Figure S6 — Correlation analysis of expression levels and the stomatal aperture of P2-type ATPases genes under four abiotic stresses. [file Image_6.TIF]
